# Structural and Functional Brain Remodeling during Pregnancy with Diffusion Tensor MRI and Resting-State Functional MRI

**DOI:** 10.1371/journal.pone.0144328

**Published:** 2015-12-10

**Authors:** Russell W. Chan, Leon C. Ho, Iris Y. Zhou, Patrick P. Gao, Kevin C. Chan, Ed X. Wu

**Affiliations:** 1 Laboratory of Biomedical Imaging and Signal Processing, The University of Hong Kong, Pokfulam, Hong Kong SAR, China; 2 Department of Electrical and Electronic Engineering, The University of Hong Kong, Pokfulam, Hong Kong SAR, China; 3 UPMC Eye Center, Ophthalmology and Visual Science Research Center, Department of Ophthalmology, University of Pittsburgh, Pittsburgh, PA, United States of America; Institute of Psychology, Chinese Academy of Sciences, CHINA

## Abstract

Although pregnancy-induced hormonal changes have been shown to alter the brain at the neuronal level, the exact effects of pregnancy on brain at the tissue level remain unclear. In this study, diffusion tensor imaging (DTI) and resting-state functional MRI (rsfMRI) were employed to investigate and document the effects of pregnancy on the structure and function of the brain tissues. Fifteen Sprague-Dawley female rats were longitudinally studied at three days before mating (baseline) and seventeen days after mating (G17). G17 is equivalent to the early stage of the third trimester in humans. Seven age-matched nulliparous female rats served as non-pregnant controls and were scanned at the same time-points. For DTI, diffusivity was found to generally increase in the whole brain during pregnancy, indicating structural changes at microscopic levels that facilitated water molecular movement. Regionally, mean diffusivity increased more pronouncedly in the dorsal hippocampus while fractional anisotropy in the dorsal dentate gyrus increased significantly during pregnancy. For rsfMRI, bilateral functional connectivity in the hippocampus increased significantly during pregnancy. Moreover, fractional anisotropy increase in the dentate gyrus appeared to correlate with the bilateral functional connectivity increase in the hippocampus. These findings revealed tissue structural modifications in the whole brain during pregnancy, and that the hippocampus was structurally and functionally remodeled in a more marked manner.

## Introduction

Mammalian females, from rodents to primates, undergo fundamental behavioral changes during pregnancy [1, 2]. Before pregnancy, female mammals are largely self-directed species that satisfy their own needs for survival. During pregnancy, they become focused on the care and well-being of their future offspring [1, 2]. Previous studies have reported that pregnancy-induced behavioral changes are associated with the hippocampal functions. For example, improvements in learning and memory and enhancement in object recognition and placement during pregnancy are related to functions of dorsal hippocampus whereas reduction in stress responsiveness and anxiety is related to functions of the ventral hippocampus [1, 3–6]. These pregnancy-induced behavioral changes may be associated with reproductive hormonal changes [7]. Estrogen and progesterone are produced in the ovaries and placenta during pregnancy, whereas prolactin and oxytocin are secreted by the hypothalamus and pituitary gland. These hormonal changes have been previously shown to remodel the brain at the neuronal level [1]. For example, estrogen and progesterone can increase dendritic spine density and neuronal excitability in the hippocampus, particularly the dentate gyrus [1, 8–11]. Prolactin can enhance white matter regeneration in the brain, and may mediate neurogenesis in the forebrain [12–14]. Oxytocin increases the firing of inhibitory hippocampal neurons, and may enhance hippocampal spike transmission [15, 16].

Despite the above findings, the effects of pregnancy on brain at the tissue level remain largely unknown. In particular, it is unclear whether pregnancy remodels the hippocampus structurally and functionally. This lack of such knowledge is partly due to limited non-invasive tools available to assess different brain tissues longitudinally and quantitatively in the living brains. Using small water molecules as a ubiquitous marker, MR diffusion tensor imaging (DTI) provides an unprecedented and quantitative capability to probing tissue microstructures noninvasively [17–19]. On the other hand, resting-state functional MRI (rsfMRI) can map and assess the functional connectivity between various brain regions based on the slow but temporally coherent blood-oxygenation-dependent MR signal fluctuations [20–23]. Together, these two in vivo methods can probe and quantify structural and functional brain changes within a large field of view across time. In this study, we applied DTI and rsfMRI to a rat pregnancy model to study the structural and functional remodeling of the brain during pregnancy. The rats were monitored at three days before mating (baseline) and seventeen days after mating (G17), with G17 equivalent to the early stage of the third trimester (29^th^ to 33^rd^ weeks of gestation) in humans. Age-matched non-pregnant controls were scanned at the same time-points. The global tissue structural changes, together with the functional connectivity changes in the hippocampus, were investigated.

## Materials and Methods

### Animal Preparation

This study was carried out in strict accordance with the recommendations in the Guide for the Care and Use of Laboratory Animals of the National Institutes of Health. The protocol was approved by the animal care and use committee of The University of Hong Kong (Permit Number: 2041–09 and 3139–13). All MRI experimental procedures were performed under isoflurane anesthesia, and all efforts were made to minimize suffering. Twelve-week old female Sprague-Dawley rats (250-280g, N = 22) were housed under a 12/12 hour light/dark cycle in a temperature controlled room with ad libitum access to food and water [24–26]. Pregnant primiparous rats (n = 15) were scanned longitudinally at three days before mating (baseline) and seventeen days after mating (G17). Age-matched nulliparous female rats (n = 7) served as non-pregnant controls and were examined at the same time-points as the pregnant rats [25].

### MRI Protocols

All MRI experiments were performed using a 7T Bruker scanner (70/16 PharmaScan, Bruker Biospin GmbH, Germany). After isoflurane induction at 3%, 1–2 drops of 2% lidocaine were applied to the chords to provide local anesthesia before endotracheal intubation. The rats were maintained at 1–1.5% isoflurane and mechanically ventilated at 54–56 cycles/min in room-temperature air using a ventilator (TOPO, Kent Scientific Corp., Torrington, CT). During MRI, the rats were placed on a plastic cradle with the head fixed with a tooth bar and plastic screws in the ear canals. Body temperature was maintained using a water circulation system. Continuous physiological monitoring was performed using an MRI-compatible system (SA Instruments, Stony Brook, NY). Vital signs were maintained within normal physiological ranges (rectal temperature 36.5–37.5°C, heart rate 350–420 beats/min, 54–56 breathes/min, and oxygen saturation >95%) throughout the experiment [24, 26–28].

Scout T_2_-weighted images were obtained using a rapid acquisition with relaxation enhancement (RARE) sequence to determine the transverse, coronal and sagittal planes of the brain. Ten slices were positioned in the transverse orientation according to the rat brain atlas [29]. For DTI, diffusion-weighted images, together with 5 images without diffusion sensitization (*b*_*0*_ images), were acquired using a 4-shot spin-echo echo-planar-imaging sequence with TR/TE = 3000/32ms, FOV = 32×32mm^2^, matrix = 128×128, slice thickness/gap = 1/0mm, 4 repetitions, Δ/δ = 5/17ms, and 30 different diffusion directions at b-value = 1000 s/mm^2^ [27, 28, 30]. For rsfMRI, a single shot gradient-echo echo-planar-imaging sequence was used with TR/TE = 1000/18ms, FOV = 32×32mm^2^, matrix = 64×64, 10 slices with slice thickness/gap = 1/0mm. A total of 420 volumes were acquired in each scanning trial, and 3 to 4 trials were obtained for each animal [24, 26]. High-resolution RARE T_2_-weighted images were acquired in the same geometric locations as DTI and rsfMRI as an anatomical reference with TR/TE = 4200/36 ms and matrix = 256×256.

### Data Analysis–DTI

Diffusion-weighted images were first registered to the respective *b*_*0*_ image using AIRv5.25 (Roger Woods, UCLA, USA), and images with severe ghosting were excluded. Mean diffusivity (MD), fractional anisotropy (FA), axial diffusivity (AD), and radial diffusivity (RD) maps were calculated [27, 28, 31]. Using SPM8 (Wellcome Department of Imaging Neuroscience, University College, London, UK), the T_2_-weighted images from individual rats were co-registered to a customized reference brain template (Figure A in S1 File) with a 3D rigid-body transformation and the resulting transforming matrix was then applied to register the respective DTI index maps.

The global changes of MD, FA, AD, and RD in the whole brain (WB), gray matter (GM), and white matter (WM) were measured. The averaged MD, FA, AD, and RD maps were calculated, and were used to define the WB, GM and WM masks [32]. Specifically, GM and WM masks were defined by voxels with MD < 1.0μm^2^/ms and 0.05 < FA < 0.25, and MD < 1.0μm^2^/ms and 0.32 < FA, respectively (Figure B in S1 File) [32]. Subsequently, histograms were plotted for each rat in all DTI index maps and the expected value of each histogram was calculated [32]. Results were compared between the baseline and G17 using two-way ANOVA, followed by post-hoc Bonferroni’s test, to separate the effects caused by development and pregnancy. Paired t-test was also applied to compare the measurements between the baseline and G17 (Figure E in S1 File).

Regionally, changes of MD, FA, AD, and RD in the hippocampus were measured. To identify the specific subregions exhibiting DTI index value changes during pregnancy, voxel-based analysis was applied to the pregnancy group by utilizing voxel-wise paired t-test for MD and FA maps with SPM8 before and after pregnancy in the pregnant primiparous rats, followed by multiple testing correction via false discovery rate [33]. Voxels with significant change (p < 0.05) at a clustering level of 6 or more voxels were defined as hot voxels. Regions of interest (ROIs) were defined in the pregnancy group according to the rat brain atlas and hot pixels. They were found to center around the dorsal hippocampus and dorsal dentate gyrus. The average regional DTI index values were obtained by averaging the DTI index values in the ROIs [33]. For the control group, the same ROIs were used. To offset the effects of global GM changes on the hippocampus changes, the hippocampal MD, FA, AD, and RD measurements were normalized with the respective global GM changes by:

$$Index_{normalized}=\frac{Index_{ROI}}{\left(1+Index_{\%\;GM\;Change}\right)}$$ (1)

Two-way ANOVA was applied to compare the measurements between the baseline and G17, followed by post-hoc Bonferroni’s test. Paired t-test was also applied to compare the measurements between the baseline and G17 (Figures F and G in S1 File).

### Data Analysis–rsfMRI

For each rsfMRI session, all images were first corrected for slice timing differences with SPM8 and then realigned to the mean image of the series using 2D rigid-body transformation. The first 20 image volumes of each session were discarded to eliminate possible non-equilibrium effects. Voxel-wise linear detrending with least-squares estimation was performed temporally to eliminate the drift caused by physiological noises and system instability. A temporal band-pass filter (0.005–0.1Hz) was applied without spatial smoothing [23, 26, 34–36]. Trials with excessive motion were excluded, resulting in an average of 3.32 trials per rat for subsequent analysis (Table A in S1 File). Inter-animal co-registration was performed with SPM8 using similar procedures as DTI image co-registration described above.

To determine whether the bilateral rsfMRI connectivity alters in the hippocampus during pregnancy, both seed-based analysis (SBA) and independent component analysis (ICA) were performed. For SBA, a 2×2-voxel region was chosen as the seed in the hippocampus [20, 23]. Regionally averaged time course from the voxels within the seed served as the reference time course. Pearson’s correlation coefficients were calculated between the reference time course and the time course of each voxel, and a 2×2-voxel region on the contralateral side of the seed was defined as the ROI (Figure C1 in S1 File). Mean correlation coefficients were obtained from averaging the correlation coefficients within the ROI. The procedure was repeated with the seed and ROI switched, and the two mean correlation coefficient values were averaged.

For ICA, co-registered rsfMRI data were analyzed with GIFT v2.0d Toolbox [37–39]. In brief, 37 components were selected for the Infomax algorithm and group-level ICA was performed on all rsfMRI data from the same time-point [26]. The group-level spatial ICA maps of the resting-state networks were scaled to z-scores, and were visually inspected. The bilateral hippocampal network was identified based on the spatial patterns with reference to known anatomical and functional locations in the rat brain atlas [29, 34]. The ROI was defined in the hippocampus based on the rat brain atlas (Figure C2 in S1 File), and the average z-score were obtained by regional averaging in the ROI. Two-way ANOVA was applied to compare the SBA and ICA measurements between baseline and G17, followed by post-hoc Bonferroni’s test. Paired t-test was also applied to compare the measurements between the baseline and G17 (Figure H in S1 File).

## Results

### Brain Global Structural Changes during Pregnancy

**Fig 1:**
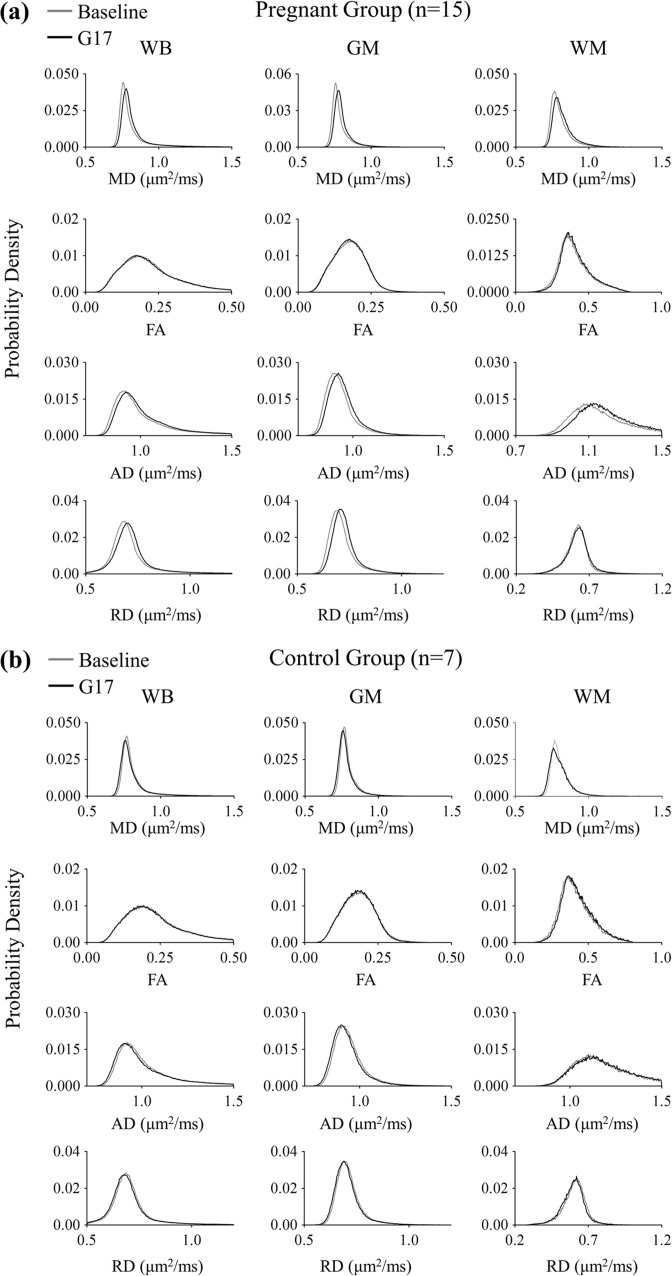
Histogram comparison of the tissue structural changes in the global brain between three days before (baseline) and seventeen days after mating (G17) measured by DTI in the pregnancy (a) and control (b) groups. Mean diffusivity (MD), fractional anisotropy (FA), axial diffusivity (AD) and radial diffusivity (RD) were evaluated in the whole brain (WB), gray matter (GM) and white matter (WM). They were generally observed to increase during pregnancy **(a)**. In contrast, slight decreases were seen in the control group **(b)**.

**Fig 2:**
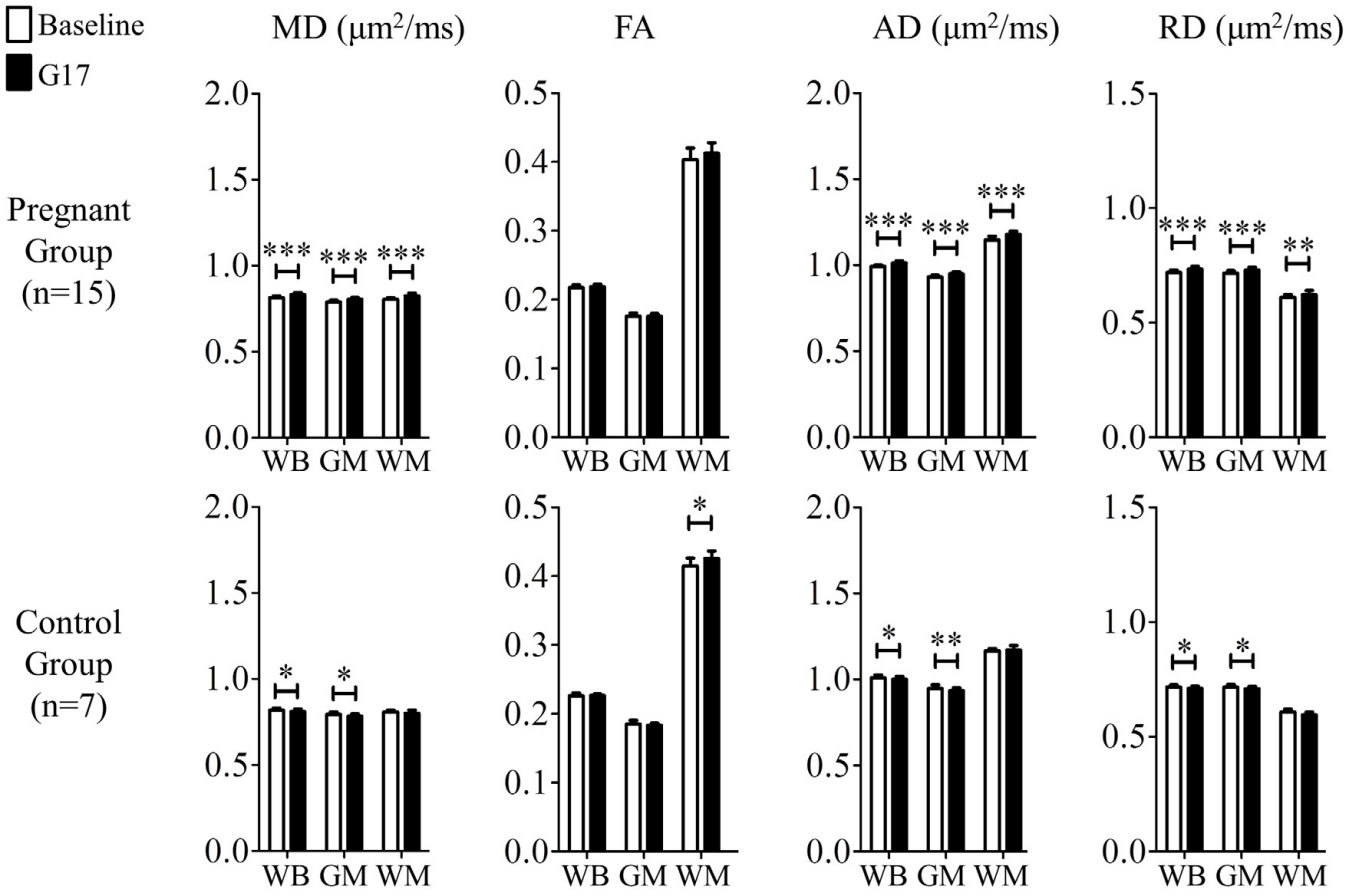
The comparison of the tissue structural changes in the global brain between the baseline and G17 in the pregnancy (top) and control (bottom) groups. In the pregnancy group, MD, AD and RD increased globally, indicating tissue microstructural remodeling (that facilitated water molecular diffusion) in the global brain. Two-way ANOVA was applied, followed by post-hoc Bonferroni’s test. *, ** and *** denote p<0.05, p<0.01 and p<0.001, respectively. Error bars indicate the standard deviation.

Fig 1 shows the results of the histogram analyses of all DTI index measurements in both pregnancy and control groups. In general, all diffusivities including MD, AD and RD increased in the WB, GM and WM during pregnancy. Fig 2 summarizes the DTI index changes in the WB, GM, and WM between the baseline and G17. In the pregnancy group, diffusivities were observed to generally increase. Overall, the MD, AD and RD in the WB increased by 2.0 ± 0.3%, 1.8 ± 0.3% and 1.8 ± 0.3% (mean ± standard error of mean, Bonferroni’s post-hoc test, p<0.001), respectively. Similar results were observed in the GM, with percentage increase of 1.7 ± 0.3%, 1.7 ± 0.3% and 1.8 ± 0.3% (Bonferroni’s post-hoc test, p<0.001) in MD, AD and RD, respectively. In the WM, the percentage increase in MD and AD were larger than those in RD. They were 2.6 ± 0.4% (Bonferroni’s post-hoc test, p<0.001), 3.1 ± 0.5% (Bonferroni’s post-hoc test, p<0.001) and 1.7 ± 0.5% (Bonferroni’s post-hoc test, p<0.01), respectively. In addition, FA was observed to have a tendency to increase, exhibiting 3.0 ± 1.1% in the WM (and 0.8 ± 0.2% in the WB). In the control group, all diffusivities showed slight decreases (i.e., 0.8 ± 0.2% MD decrease in the WB) while FA exhibited 2.7 ± 1.0% increase as expected due to the brain developmental maturation over the 3-week observation period [27, 28]. All together, these results demonstrated the general brain diffusivity increases during pregnancy, directly revealing tissue structural changes in the global brain.

### Structural Changes in the Hippocampus during Pregnancy

**Fig 3:**
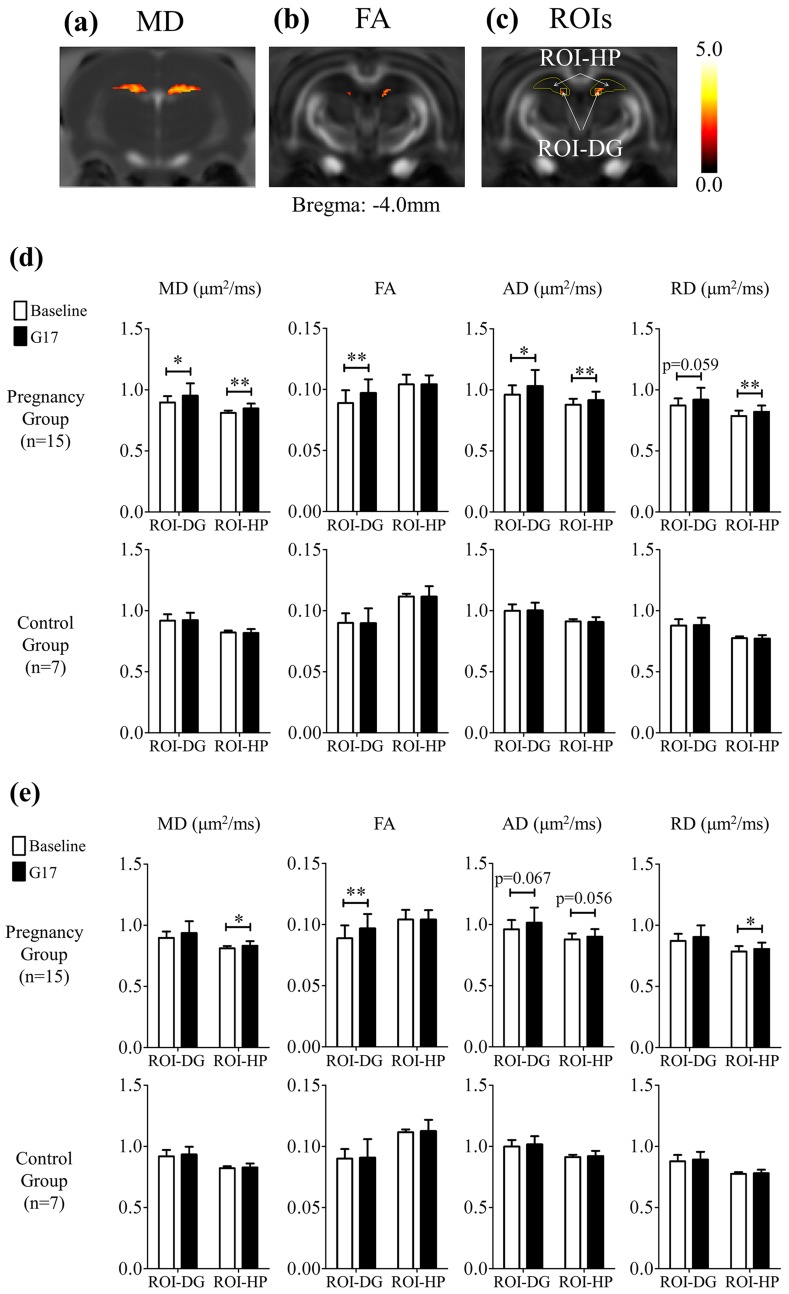
The results of the voxel-based analysis followed by multiple testing corrections via false discovery rate in MD (a) and FA (b) of the pregnancy group. The hot voxels indicated significant increase during pregnancy, and the threshold applied was p = 0.05. With reference to (a), (b) and the rat brain atlas, regions of interest (ROIs) were defined (c) and employed for quantitative analysis. The ROI-HP covers the dorsal hippocampus, and the ROI-DG covers the dorsal dentate gyrus (DG). (d) The summary of the local tissue structural changes between the baseline and G17 in the ROI-DG and ROI-HP of both the pregnancy and control groups without any normalization. There was significant increase in FA in the ROI-DG, and significant increase in MD, AD and RD in the ROI-HP during pregnancy, but not in the controls. After normalization with global GM changes (e), MD, AD and RD changes in ROI-HP became smaller but remained increased, while the increase in FA in the ROI-DG was similar. These results indicated the presence of more pronounced tissue structural remodeling in the dorsal hippocampus, including the dorsal dentate gyrus during pregnancy. Two-way ANOVA was applied, followed by post-hoc Bonferroni’s test. * and ** denote p<0.05 and p<0.01 and, respectively. Error bars indicate the standard deviation.

Fig 3A and 3B shows the results of voxel-based analysis of MD and FA changes, respectively, in the hippocampus. Two ROIs were then defined according to the rat brain atlas and the results of voxel-based analysis (Fig 3C). The first ROI covered the dorsal hippocampus (ROI-HP), and the second one consisted of two mirrored squares covering the dorsal dentate gyrus (ROI-DG). Fig 3D summarizes MD, FA, AD and RD changes in these two ROIs. The results were normalized with global GM changes, and subsequently summarized in Fig 3E. Before normalization, the MD, AD and RD in ROI-HP increased by 4.9 ± 1.3%, 4.8 ± 1.3% and 5.0 ± 1.2% (Bonferroni’s post-hoc test, p<0.01), respectively, during pregnancy but not in the controls, whereas FA in ROI-DG increased by 9.5 ± 2.0% (Bonferroni’s post-hoc test, p<0.01) during pregnancy but not in the controls. With normalization, the increases of MD, AD and RD in ROI-HP were 3.2 ± 1.2% (Bonferroni’s post-hoc test, p<0.05), 3.1 ± 1.2% (Bonferroni’s post-hoc test, p = 0.056), and 3.3 ± 1.2% (Bonferroni’s post-hoc test, p<0.05), respectively, during pregnancy, whereas FA increase in ROI-DG remained significant and was 9.6 ± 2.2% (Bonferroni’s post-hoc test, p<0.01). These results indicated the presence of more pronounced tissue structural remodeling in the dorsal hippocampus, including the dorsal dentate gyrus during pregnancy.

### Functional Connectivity Changes in Bilateral Hippocampi during Pregnancy

**Fig 4:**
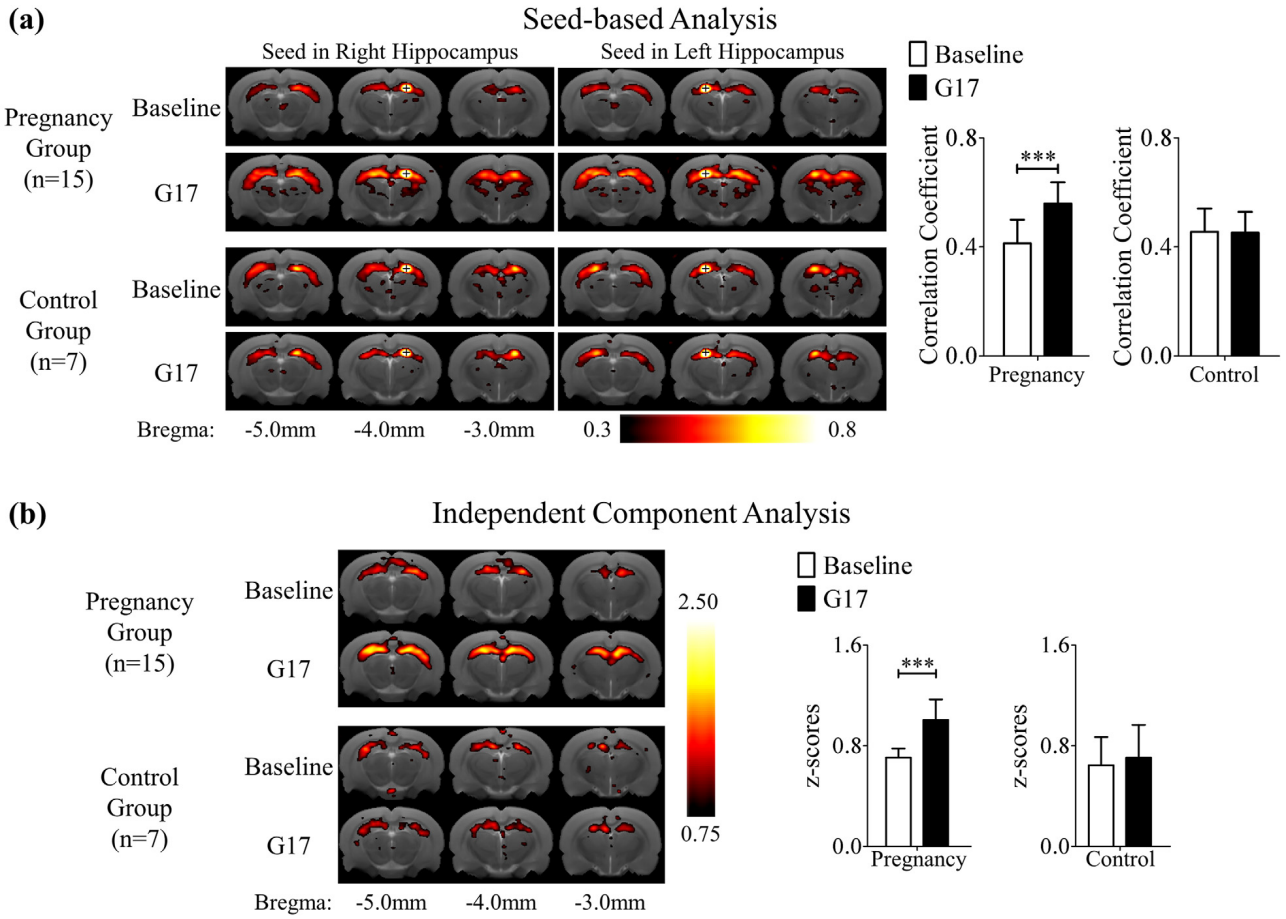
(a) The mean correlation coefficient maps of rsfMRI obtained using seed based analysis (SBA) with seeds (crosses) in the right or left dorsal hippocampus. (b) The mean z-score maps obtained using independent component analysis (ICA). The mean correlation coefficient maps and mean z-score maps demonstrate the presence of the bilateral hippocampal rsfMRI connectivity in both pregnancy and control groups at baseline and G17. Both types of connectivity maps show that the rsfMRI connectivity in the hippocampus became stronger during pregnancy. These results indicated that the bilateral hippocampus became more functionally connected during pregnancy. The maps are overlaid on T2-weighted anatomical image. Two-way ANOVA was applied followed by post-hoc Bonferroni’s test. *** denotes p<0.001. Error bars indicate the standard deviation.

Fig 4A and 4B shows the results of SBA and ICA, respectively in the hippocampus. The mean correlation coefficient maps and z-score maps demonstrate the presence of the bilateral hippocampal rsfMRI connectivity in both pregnancy and control groups at baseline and G17. More importantly, both maps show that the rsfMRI connectivity in the hippocampus became stronger during pregnancy. Quantitatively, the correlation coefficient obtained in SBA increased significantly by 38.8 ± 6.2% (Bonferroni’s post-hoc test, p<0.001) in the hippocampus and the z-score obtained in ICA increased significantly by 43.9 ± 7.2% (Bonferroni’s post-hoc test, p<0.001) during pregnancy. Note that similar ICA analysis was also performed for the bilateral primary and secondary somatosensory networks, revealing no significant connectivity strength changes in both pregnancy and control groups between baseline and G17 (Figure D in S1 File). These results indicated that the bilateral hippocampus became more functionally connected during pregnancy.

### Structural vs. Functional Changes in Hippocampal Tissues

**Fig 5:**
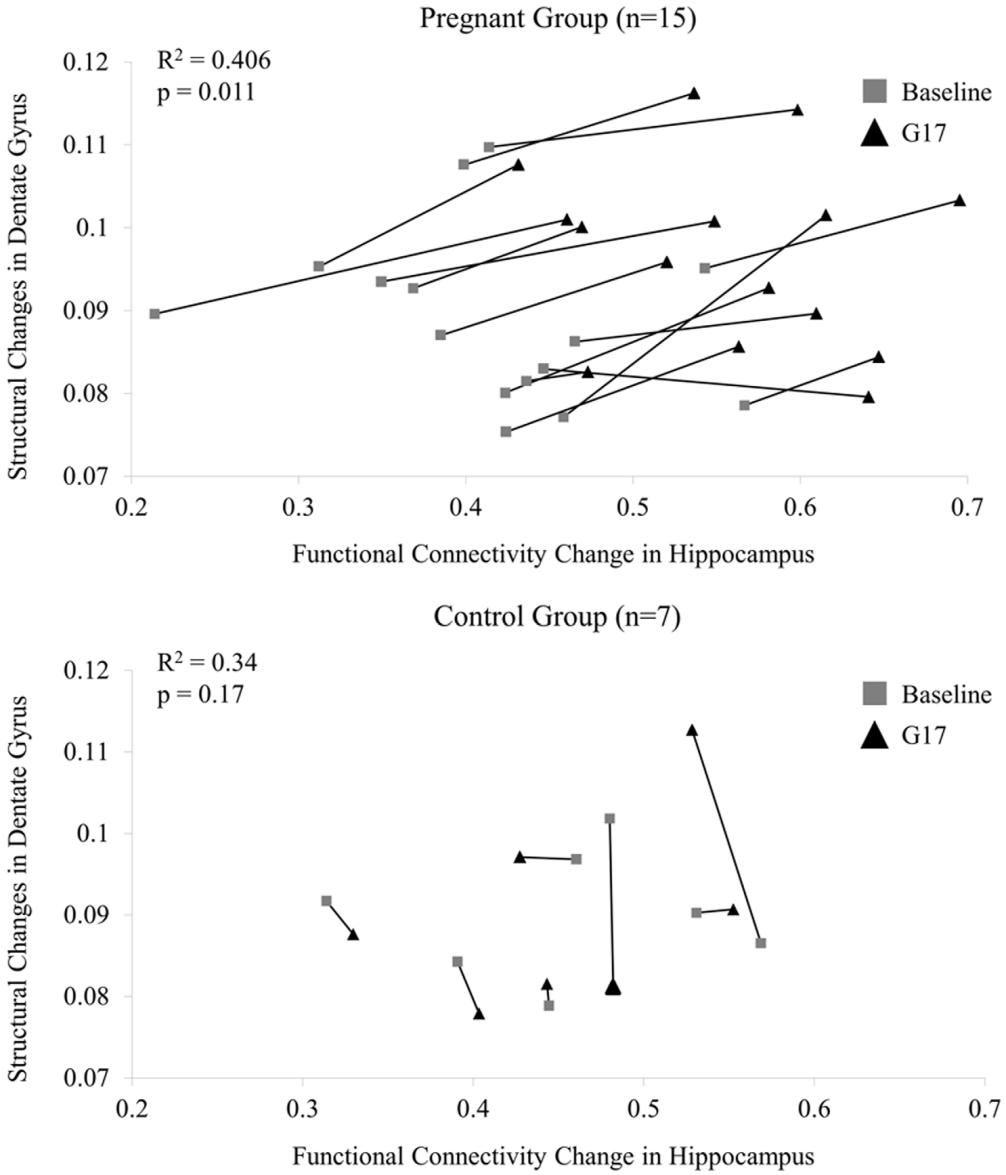
The relationship between FA in dorsal dentate gyrus and functional connectivity in hippocampus of individual rats from baseline to G17 in the pregnancy group (upper panel) and control group (lower panel). These results indicated that DTI-based structural changes in the dorsal dentate gyrus and the bilateral rsfMRI connectivity changes in the hippocampus during pregnancy were correlated and might be coupled. In contrast, no such correlation was observed in the control group.

The scatter plots in Fig 5 display the relationships between the FA changes in the dorsal dentate gyrus (ROI-DG in Fig 3) and the bilateral rsfMRI connectivity changes in the hippocampus of individual rats in both pregnancy and control groups. These DTI and rsfMRI changes were found to correlate in the pregnancy group (R^2^ = 0.406; p = 0.011) but not in the control group (R^2^ = 0.34; p = 0.17). Presence of such MD and FA correlation in the pregnancy group suggested a coupling between structural and functional changes in the hippocampus during pregnancy.

## Discussion

### Structural Changes in the Global Brain

Diffusivity is sensitive to various cellular-level tissue microstructures that modulate the extent and behavior of water molecular diffusion. The global diffusivity increases observed between the baseline and G17 in this study (Figs 1 and 2) revealed that the brain tissue microstructure became more diffusion-friendly or permeable to water molecular diffusion movements during pregnancy. Thus they directly indicated the tissue structural changes in the global brain during pregnancy. The mechanisms underlying such global tissue changes at microscopic levels as probed by in vivo DTI can be multi-faceted and complex. One possibility is that such diffusivity increase may be closely associated with the decrease of taurine concentration in the brain and/or the increase of extracellular fluid. Taurine is an organic acid in the central nervous system that prevents neurons and glial cells from shrinking. Its concentration in the brain has been found to decrease during pregnancy, which can cause neuron and glial cell shrinkage [25, 40–43]. Consequently, extracellular space may increase during pregnancy. In fact, extracellular fluid has been reported to increase in the brain during pregnancy [44], and the increase in extracellular fluid has been shown to well correlate with the increase of tissue apparent diffusion coefficient (ADC) or MD because more extracellular space presents lesser restriction of water molecule diffusion [45–49]. All together, the cell shrinkage and increase of extracellular space/fluid likely contributed to the global diffusivity increases during pregnancy observed in the present study.

### Structural Changes in the Hippocampus

The increase of normalized MD in the dorsal hippocampus during pregnancy (Fig 3) indicated that tissue structural changes in these regions were more pronounced than other GM structures. Such distinctive tissue structural changes may be relevant to the functions of the dorsal hippocampus. For example, previous studies have implicated that the dorsal hippocampus is involved in memory and spatial navigation [50], and pregnancy could result in improvements in learning and memory [4], as well as enhancement in object recognition and placement [5]. Our previous MR spectroscopy study of the same rat pregnancy model revealed a pronounced decrease in taurine concentration in the hippocampus during pregnancy while other brain regions showed no detectable changes [25]. Given the key role of taurine in brain cell osmoregulation [40–42], this MR spectroscopy finding suggests that the effect of cell shrinkage may be more prominent in the hippocampus than that in other brain regions. Thus hippocampus will experience more extracellular space increase, leading to more MD increase in hippocampus than in other GM regions.

The increase of FA in the dorsal dentate gyrus was observed during pregnancy, with or without global GM normalization (Fig 3). Such structural changes may be associated with potential behavioral changes during pregnancy that involve the DG. For example, it has been demonstrated that the dorsal dentate gyrus mediates spatial pattern separation [51], which is essential for spatial learning and memory, and object recognition and placement. These behavioral functions have been shown to improve during pregnancy [4, 5]. However, the biological processes underlying such gray matter diffusion anisotropy increases are expected to be complex. They remain largely unknown in the current DTI literature though several recent studies have reported learning- or fear-induced hippocampal diffusivity changes and FA increases [33, 52, 53]. Previous electrophysiology studies demonstrated an increase in neuronal excitability in the DG during pregnancy, and the increase in excitability has been shown to correlate with the increase in dendritic spine density [8, 54]. If dendritic spine density increase in hippocampus is assumed to be spatially random, hippocampal MD should be expected to decrease due to more restricted diffusion environment. However, both dorsal hippocampal MD and DG MD were observed to increase in the present study, which might still reflect the competing outcome of the dendritic spine density increase effects vs. cell shrinkage and extracellular space expansion effects. It might also be plausible that DG dendritic spine density increase occurs anisotropically, thus yielding local FA increase as observed in the present study. Nevertheless, future biochemical studies are needed to elucidate the cellular and subcellular processes underlying the water diffusion alterations detected by DTI in hippocampus.

### Functional Connectivity Changes in Bilateral Hippocampi

rsfMRI connectivity measurements have been shown to correspond strongly with dendritic spine distribution, electrophysiological properties and behavioral quantities [55–58]. The dendritic spine density has been found to increase during pregnancy in the hippocampus [1, 11]. Electrophysiology studies showed an increase in neuronal excitability in the hippocampus during pregnancy or elevated estrogen level [8, 10]. These neuronal structural and functional changes in the hippocampus could result in an increase in bilateral rsfMRI connectivity strength observed in this study. In fact, the increase in hippocampal effective connectivity after the administration of estrogen was demonstrated in a positron emission tomography study [59], which was consistent with our present rsfMRI results. Behaviorally, the bilateral rsfMRI connectivity increase in the hippocampus could be closely associated to improved memory and learning performance during pregnancy [1, 4, 34, 60–63].

Lastly, the neurophysiological basis of resting-state connectivity has been widely stipulated to closely associate to the structural connectivity though the detailed mechanisms remain unclear [26, 64–68]. Nevertheless, the present imaging study simultaneously measured the tissue structural FA changes and functional rsfMRI connectivity changes in the hippocampus during pregnancy (Fig 5). The results suggested a coupling between the structural and functional changes measured in hippocampus among the same animals that underwent pregnancy and exhibited strong behavioral changes. Such correlation also presents direct evidence to support the close coupling between structural connectivity measured by DTI and functional connectivity measured by rsfMRI, an issue presently under debate in the neuroimaging community.

### Inter-Animal Variation in Structural and Functional Measurements

Variation between the rats was observed in both structural and functional measurements (Fig 5). In the pregnancy group, the standard deviation of FA and correlation coefficient was 0.010 and 0.086, respectively at baseline; and 0.011 and 0.077, respectively at G17. Suppose that the variation was purely contributed by experimental reproducibility and the measurements at two time-points were independent, the standard deviations of the difference between baseline and G17 should be 0.015 and 0.115, respectively, since (SD_diff_)^2^ = (SD_baseline_)^2^ + (SD_G17_)^2^_._ However, the standard deviations of the difference between the two time-points were substantially lower (i.e., 0.006 and 0.051, respectively). This indicated that experimental reproducibility issue was unlikely to be the sole contributor to the variations in Fig 5 and that the inter-animal variations in FA and correlation coefficient likely existed. Despite these inter-animal variations, Fig 5 reveals that the structural and functional remodeling in the DG/hippocampus during pregnancy might be coupled.

The inter-animal variation of FA in the dorsal DG may be attributed to the fact that the dentate gyrus is highly plastic. Previous studies have shown that neurogenesis persists in the DG of adult rodents [69], where an enriched environment and experience could increase the neurogenesis [70, 71]. Running could also increase cell proliferation and neurogenesis in the DG of mice [72]. Several DTI studies have reported changes in the DG with rats undergoing learning memory tasks [33, 53] or with rats having epilepsy [73]. In this study, the standard deviation at baseline was 0.01, which was lower than that (0.05) in the study by Parekh et al [73].

Previous studies have suggested that rsfMRI connectivity variations could be associated with individual variability in behavior [55, 74]. This variability in rsfMRI connectivity may be related to individual differences in performance [74]. Given the variability in behavior and performance among individual rats, it is not entirely surprising to observe inter-animal variability in rsfMRI connectivity [75]. Such variability may arise from the different experiences and environments during adulthood.

### Technical Considerations

Blood oxygen level dependent (BOLD) signal reflects the hemodynamic changes caused by neuronal activity, but it could be affected by other physiological changes in the brain [76]. Pregnancy may introduce physiological changes such as blood flow changes, and globally affect the BOLD signal fluctuations. ICA is a data driven analysis [37], which could potentially separate artifacts resulting from motion and/or other global physiological fluctuations such as heart rate and respiration rate. Thus, the ICA results may be interpreted as being physiologically normalized. In this study, the bilateral hippocampal rsfMRI connectivity obtained from SBA and ICA was found to be consistent (Fig 4A and 4B). They suggested that the observed increase in bilateral hippocampal rsfMRI connectivity is likely not predominated by the global physiological changes during pregnancy.

### Future Directions

Our results revealed the potential coupling between tissue structural changes and functional connectivity changes during pregnancy. However, the underlying causal mechanisms remain unclear, for which a comprehensive study of pregnancy with direct comparisons with hormonal levels and behaviors is desired in future studies. Future studies may also encompass histological examinations to correlate the observed DTI changes with cellular microstructures (such as intracellular, extracellular, neurons, glia cells and axons), membrane permeability or water exchange, and other biophysical properties associated with different water populations [27, 28].

Previous studies have reported that the cingulate cortex, hypothalamus, medial preoptic area, orbitofrontal cortices and amygdala are actively involved during pregnancy [1, 77, 78]. The present study only found the pronounced increase in the bilateral hippocampal connectivity (but not in the two other major bilateral connectivities analyzed–primary and secondary somatosensory connectivities). Other connectivities were not examined in the present study because of the technical challenges in rodent rsfMRI (i.e., relatively low SNR and spatial resolution of raw data, thus low sensitivity in detecting small connectivity changes). Future studies may employ large sample sizes and improved MRI acquisition and analysis protocol to investigate the rsfMRI connectivity changes in these brain regions.

It is also imperative to investigate whether or not the observed DTI and rsfMRI changes are transient or permanent. There are two critical stages where the brain may alter after parturition, namely, the lactation period and after weaning [1]. Since pup-induced behavioral changes exist during lactation, the structural and functional remodeling as observed in the present study may continue after G17 [79, 80]. After weaning, the estrogen level is known to normalize. Remodeling may cease; structural and functional normalization may be expected to a certain extent. Whether these expected physiological changes or the abnormalities of these physiological phenomena such as during pre-eclampsia [44, 81] can be detected by DTI or rsfMRI will be the subject of future studies.

Partial volume effect arises in volumetric images when more than one tissue type occurs in a voxel [82, 83]. It has been one of the limitations for most rodent DTI and rsfMRI investigations so far because of the relatively low spatial resolution. To achieve sufficient SNR in EPI images in this present study, the DTI and rsfMRI images were acquired with relatively low resolution. Future studies may employ recently developed diffusion methods to increase the spatial resolution and reduce or/and correct the partial volume effect [84, 85].

## Conclusion

The rat pregnancy model was longitudinally examined by in vivo DTI and rsfMRI. Diffusivities generally increased in the whole brain during pregnancy, directly documenting global tissue structural changes at microscopic levels that facilitated water molecular movement, for example, by cell shrinkage and extracellular space. Regionally, mean diffusivity in the dorsal hippocampus and fractional anisotropy in the dorsal dentate gyrus increased more pronouncedly during pregnancy but not in the control group. Bilateral rsfMRI connectivity in the hippocampus also became stronger during pregnancy but not in the control group. Moreover, fractional anisotropy increase in the dentate gyrus and the functional connectivity increase in bilateral hippocampi of the pregnancy group appeared to correlate, indicating the potential coupling of structural and functional changes in the hippocampus during pregnancy. Pregnancy remodels the brain, especially the hippocampus, both structurally and functionally.

## Supporting Information

S1 File The customized reference brain template used for inter-animal co-registration. The template was obtained by co-registering and averaging across eighteen T2-weighted naïve rat brain images (**Figure A**). The definition of whole brain (WB), gray matter (GM) and white matter (WM) masks for quantifying the global brain changes between baseline and G17 in both the pregnancy and control groups. The segmentation criteria for each mask are listed at the bottom [33] (**Figure B**). With reference to the anatomy of the hippocampus and the DTI-based local structural changes, the seed and contralateral region of interest (ROI) were defined, and used for seed-based analysis (SBA) (**Figure C1**). The ROI, defined based on the anatomy of the hippocampus, was used for quantifying the z-score changes obtained from independent component analysis (ICA) (**Figure C2**). The mean z-score maps obtained using independent component analysis (ICA). The bilateral primary somatosensory network and the bilateral secondary somatosensory network were detected at both the baseline and G17 in both the pregnancy and control groups. However, the z-scores across time-points and groups were similar within the network. These results indicated that the bilateral somatosensory rsfMRI connectivity strength remained similar before and during pregnancy. Error bars indicate the standard deviation (**Figure D**). Comparison between ANOVA followed by post-hoc Bonferroni’s test and paired t-test in the global structural changes during pregnancy (**Figure E**). Comparison between ANOVA followed by post-hoc Bonferroni’s test and paired t-test in the local structural changes in the dorsal hippocampus and dorsal dentate gyrus during pregnancy (**Figure F**). Comparison between ANOVA followed by post-hoc Bonferroni’s test and paired t-test in the normalized local structural changes in the dorsal hippocampus and dorsal dentate gyrus during pregnancy (**Figure G**). Comparison between ANOVA followed by post-hoc Bonferroni’s test and paired t-test in bilateral hippocampal functional connectivity increase during pregnancy (**Figure H**). The average trials for the pregnancy and control groups at both baseline and G17 time-points used for subsequent seed-based and independent component analyses (**Table A**).
(DOCX)
