# Supplementary material for: Structural and Functional Brain Remodeling during Pregnancy with Diffusion Tensor MRI and Resting-State Functional MRI
Source: PLoS One. 2015 Dec 10;10(12):e0144328. doi: 10.1371/journal.pone.0144328 (PMC4675543; doi:10.1371/journal.pone.0144328)
Supplement: S1 File — The template was obtained by co-registering and averaging across eighteen T2-weighted naïve rat brain images (Figure A). The definition of whole brain (WB), gray matter (GM) and white matter (WM) masks for quantifying the global brain changes between baseline and G17 in both the pregnancy and control groups. The segmentation criteria for each mask are listed at the bottom [33] (Figure B). With reference to the anatomy of the hippocampus and the DTI-based local structural changes, the seed and contralateral region of interest (ROI) were defined, and used for seed-based analysis (SBA) (Figure C1). The ROI, defined based on the anatomy of the hippocampus, was used for quantifying the z-score changes obtained from independent component analysis (ICA) (Figure C2). The mean z-score maps obtained using independent component analysis (ICA). The bilateral primary somatosensory network and the bilateral secondary somatosensory network were detected at both the baseline and G17 in both the pregnancy and control groups. However, the z-scores across time-points and groups were similar within the network. These results indicated that the bilateral somatosensory rsfMRI connectivity strength remained similar before and during pregnancy. Error bars indicate the standard deviation (Figure D). Comparison between ANOVA followed by post-hoc Bonferroni’s test and paired t-test in the global structural changes during pregnancy (Figure E). Comparison between ANOVA followed by post-hoc Bonferroni’s test and paired t-test in the local structural changes in the dorsal hippocampus and dorsal dentate gyrus during pregnancy (Figure F). Comparison between ANOVA followed by post-hoc Bonferroni’s test and paired t-test in the normalized local structural changes in the dorsal hippocampus and dorsal dentate gyrus during pregnancy (Figure G). Comparison between ANOVA followed by post-hoc Bonferroni’s test and paired t-test in bilateral hippocampal functional connectivity increase during pregnancy (Fig [file pone.0144328.s001.docx]

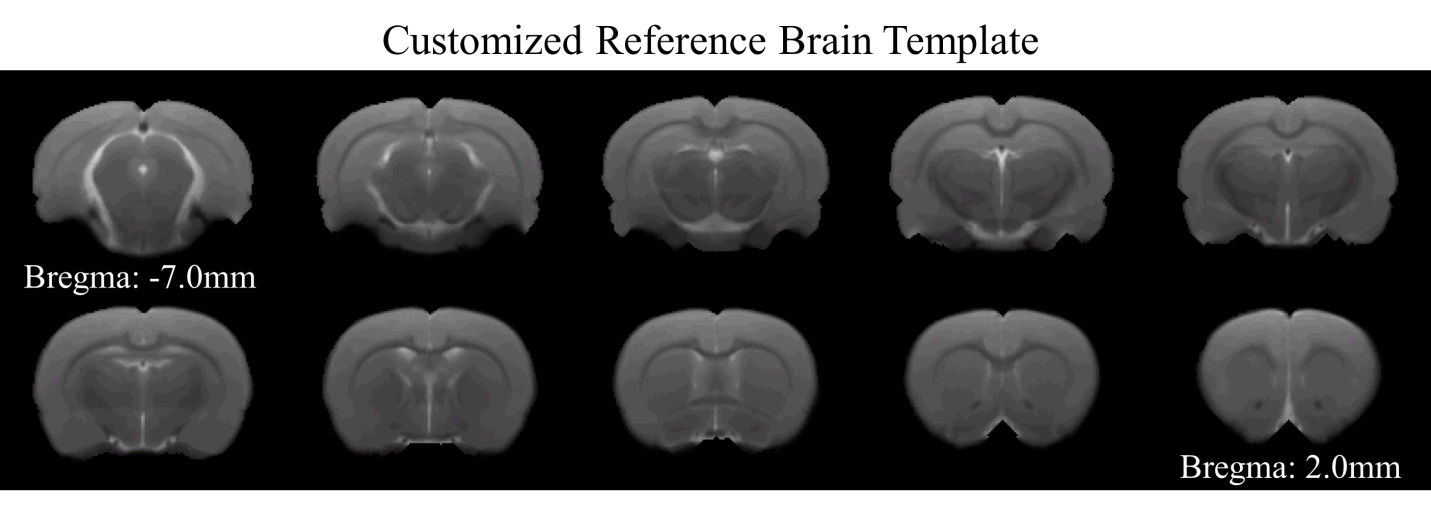


**Figure A.** The customized reference brain template used for inter-animal co-registration. The template was co-registered and averaged across eighteen T2-weighted naïve rat brain images.


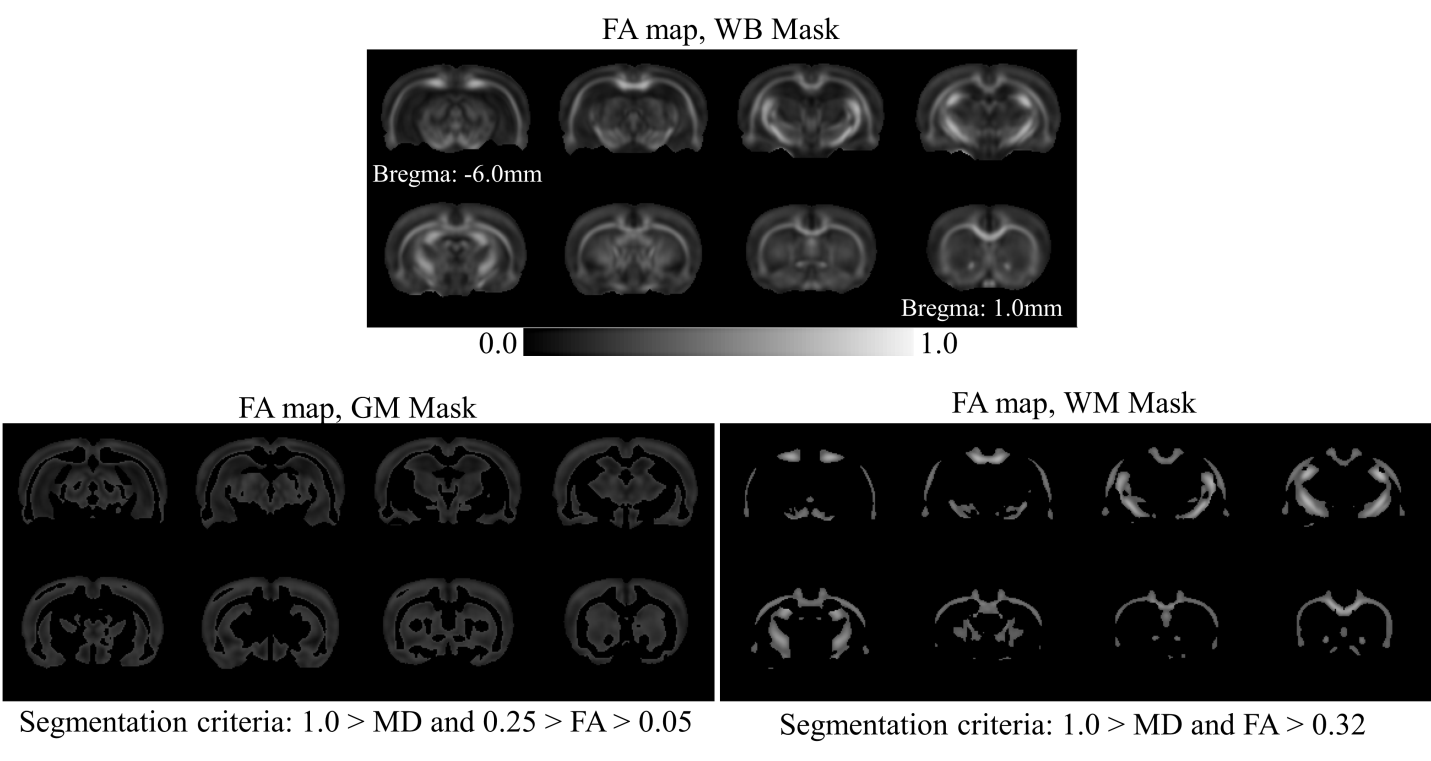


**Figure B.** The definition of whole brain (WB), gray matter (GM) and white matter (WM) masks for quantifying the global brain changes between baseline and G17 in both the pregnancy and control groups. The segmentation criteria for each mask are listed at the bottom.


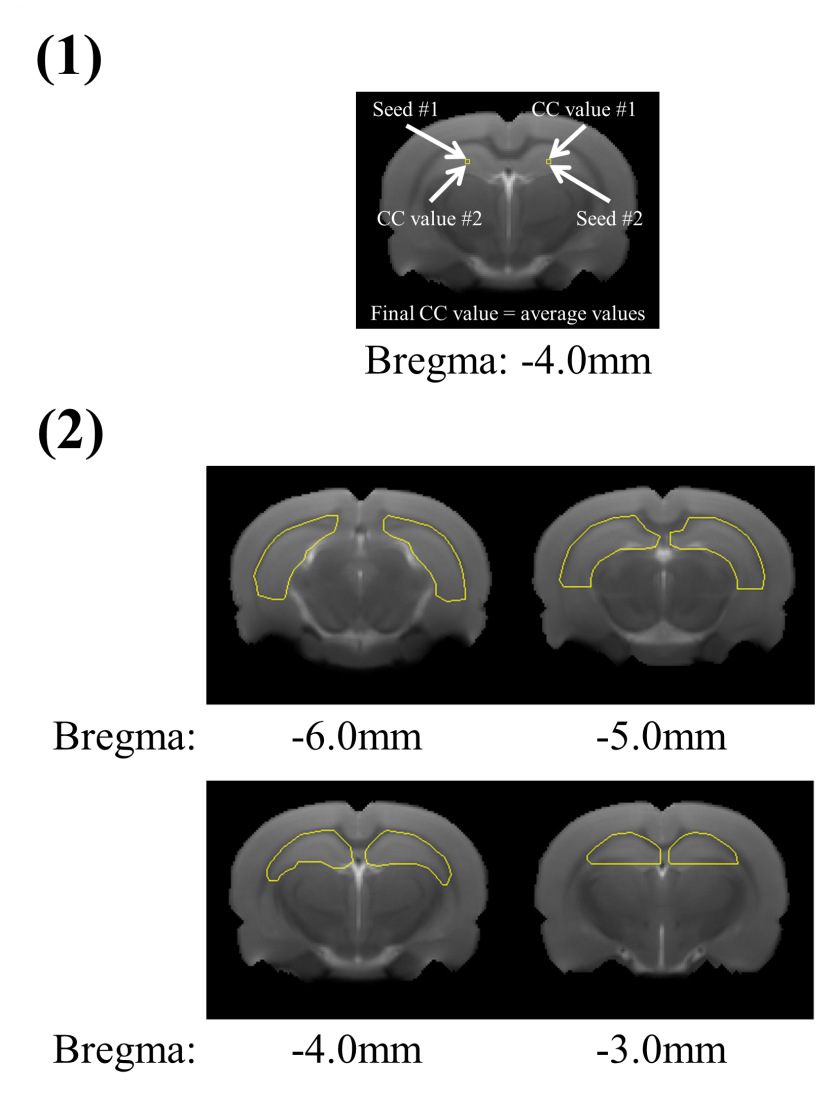


**Figure C.** **(1)** With reference to the anatomy of the hippocampus and the DTI-based local structural changes, the seed and contralateral region of interest (ROI) were defined, and used for seed-based analysis (SBA). **(2)** The ROI, defined based on the anatomy of the hippocampus, was used for quantifying the z-score changes obtained from independent component analysis (ICA).

**
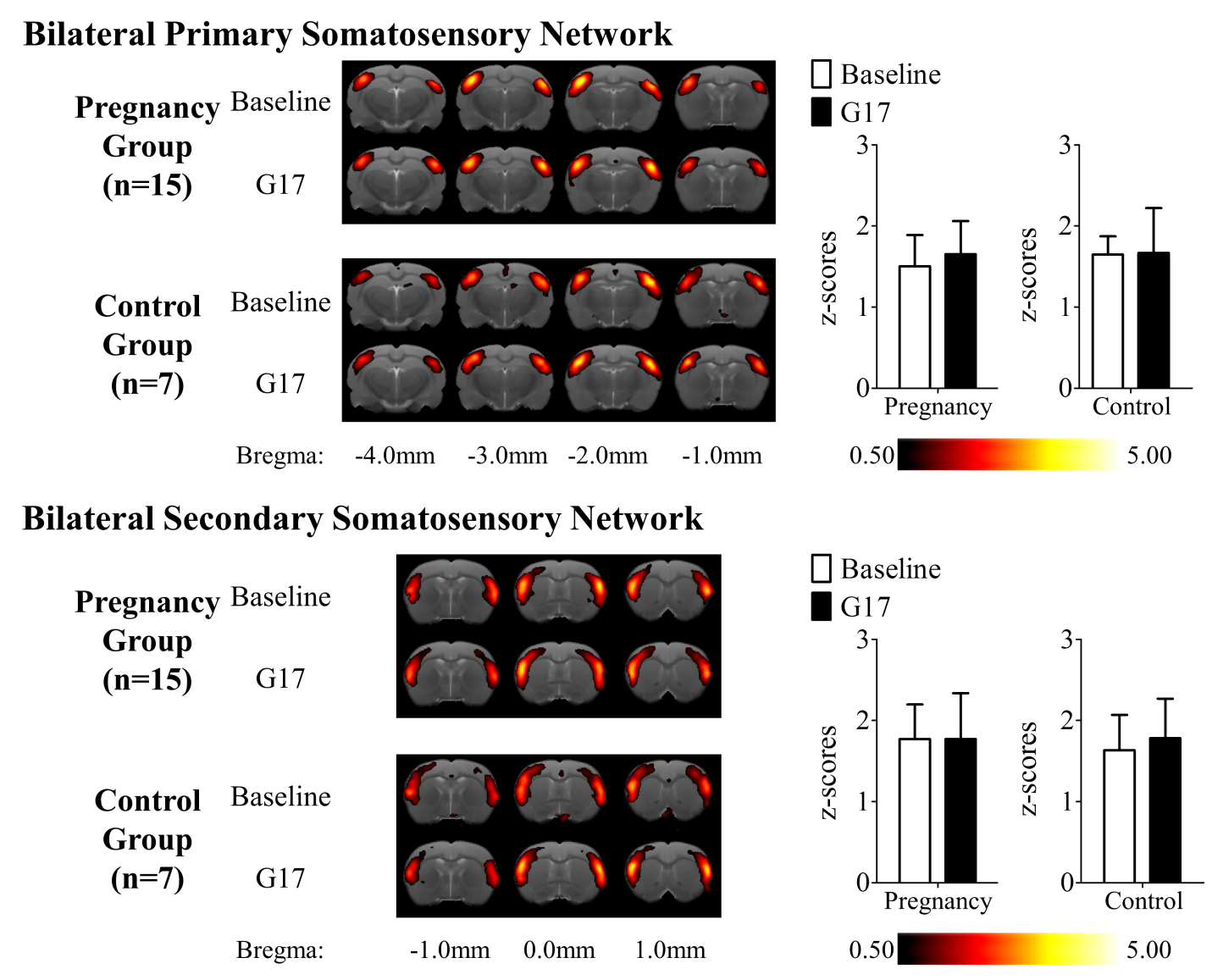
**

**Figure D.** The mean z-score maps obtained using independent component analysis (ICA). The bilateral primary somatosensory network and the bilateral secondary somatosensory network were detected at both the baseline and G17 in both the pregnancy and control groups. However, the z-scores across time-points and groups were similar within the network. These results indicated that the bilateral somatosensory rsfMRI connectivity strength remained similar before and during pregnancy. Error bars indicate the standard deviation.

**
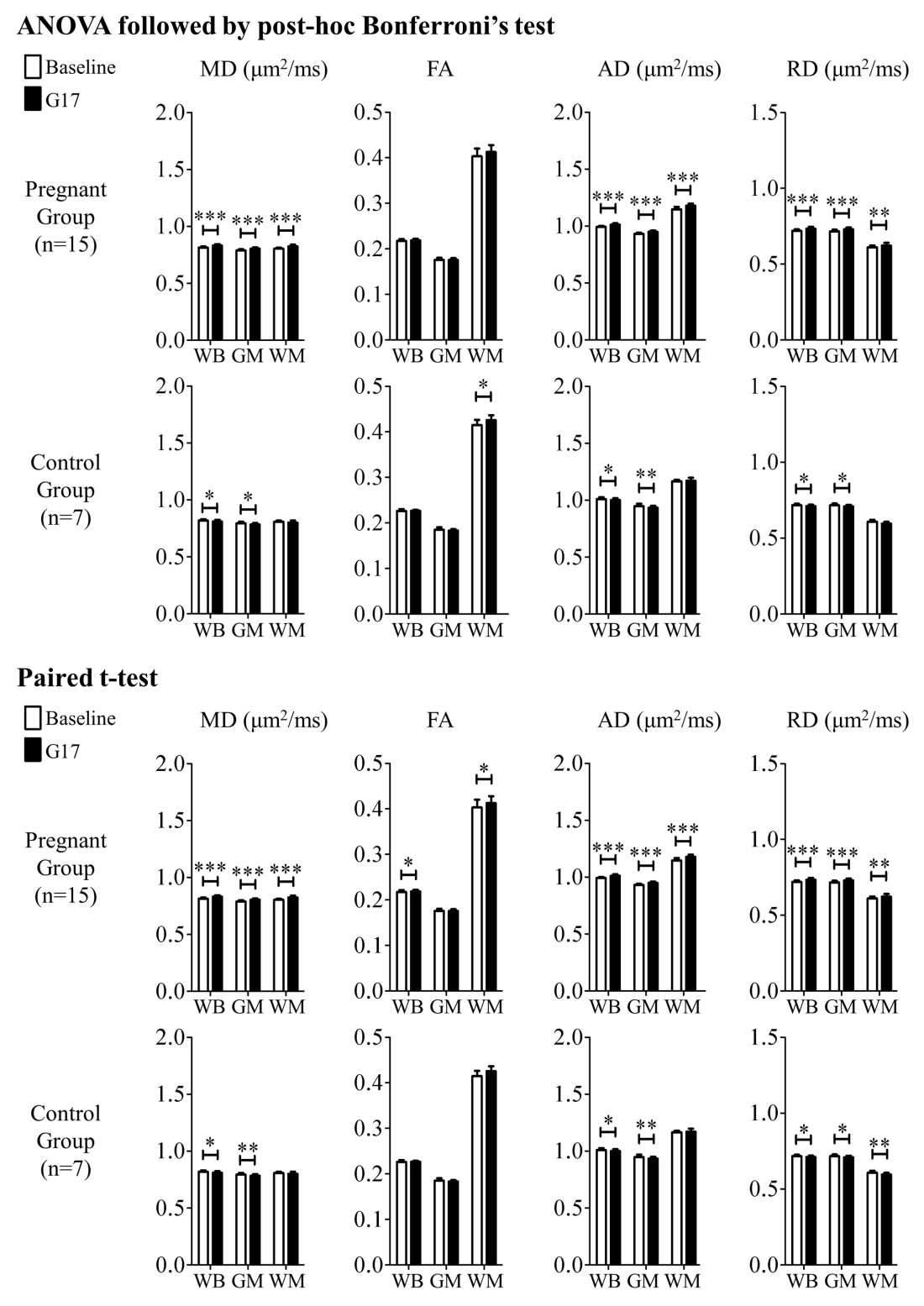
**

**Figure E.** Comparison between ANOVA followed by post-hoc Bonferroni’s test and paired t-test in the global structural changes during pregnancy.


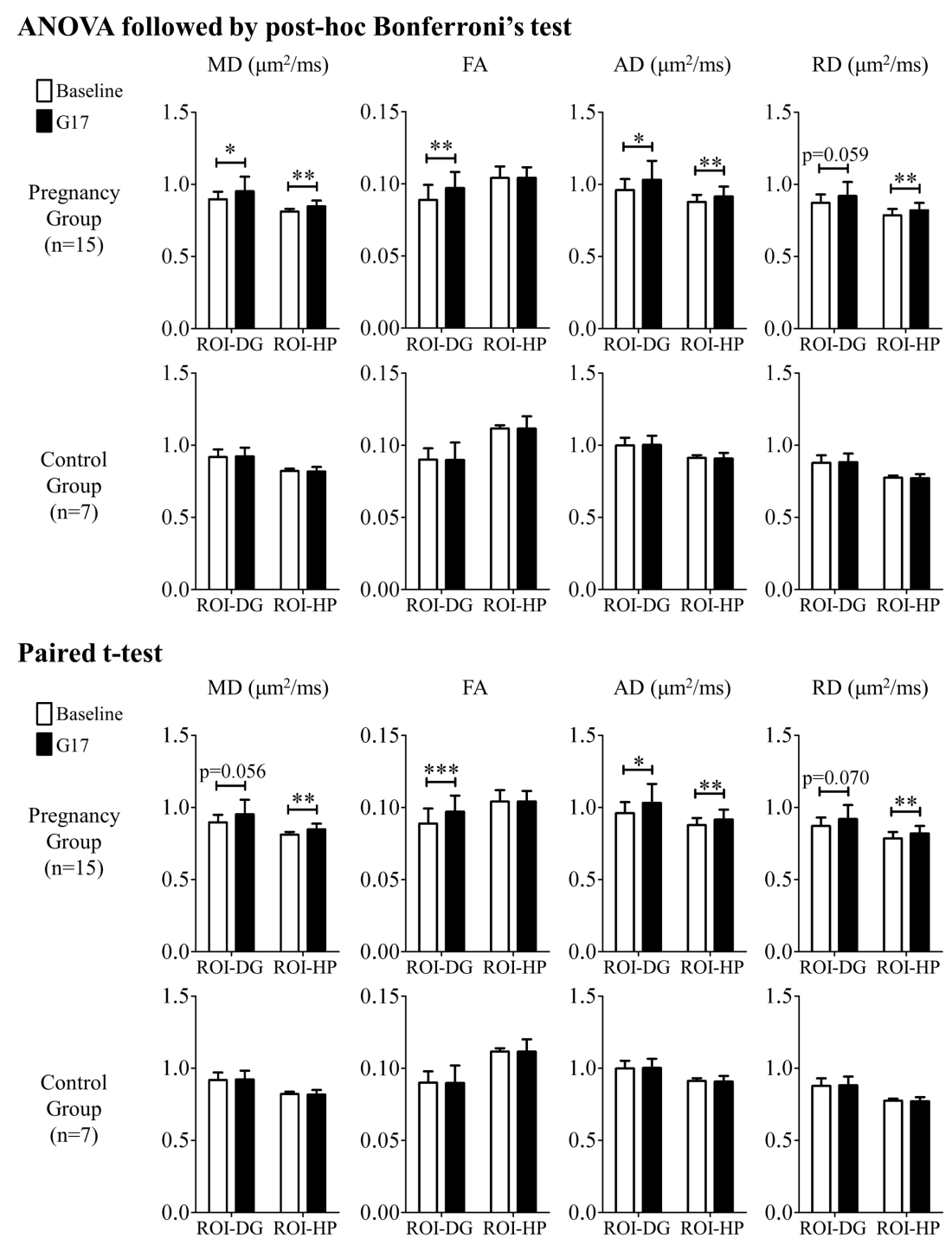


**Figure F.** Comparison between ANOVA followed by post-hoc Bonferroni’s test and paired t-test in the local structural changes in the dorsal hippocampus and dorsal dentate gyrus during pregnancy.


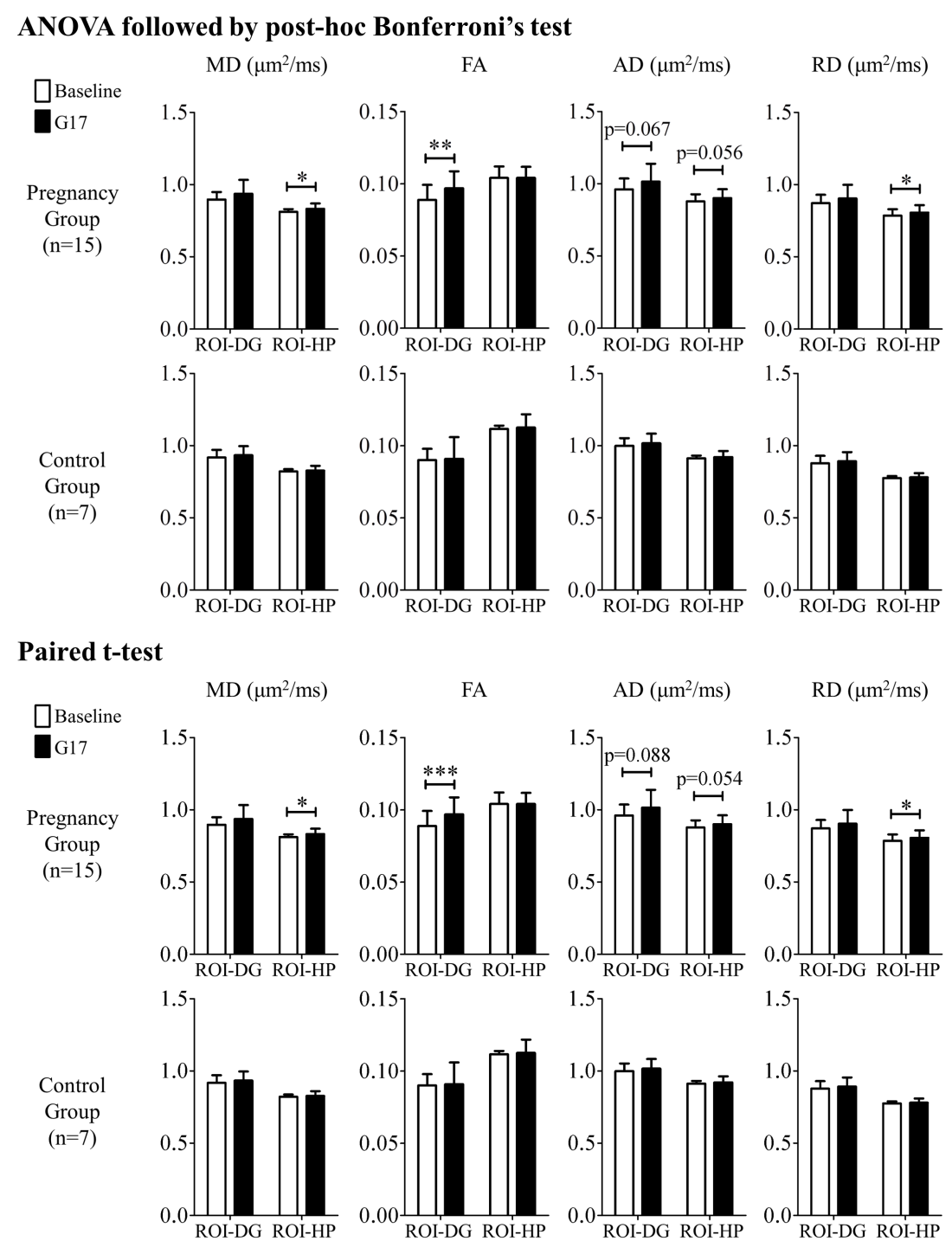


**Figure G.** Comparison between ANOVA followed by post-hoc Bonferroni’s test and paired t-test in the normalized local structural changes in the dorsal hippocampus and dorsal dentate gyrus during pregnancy.


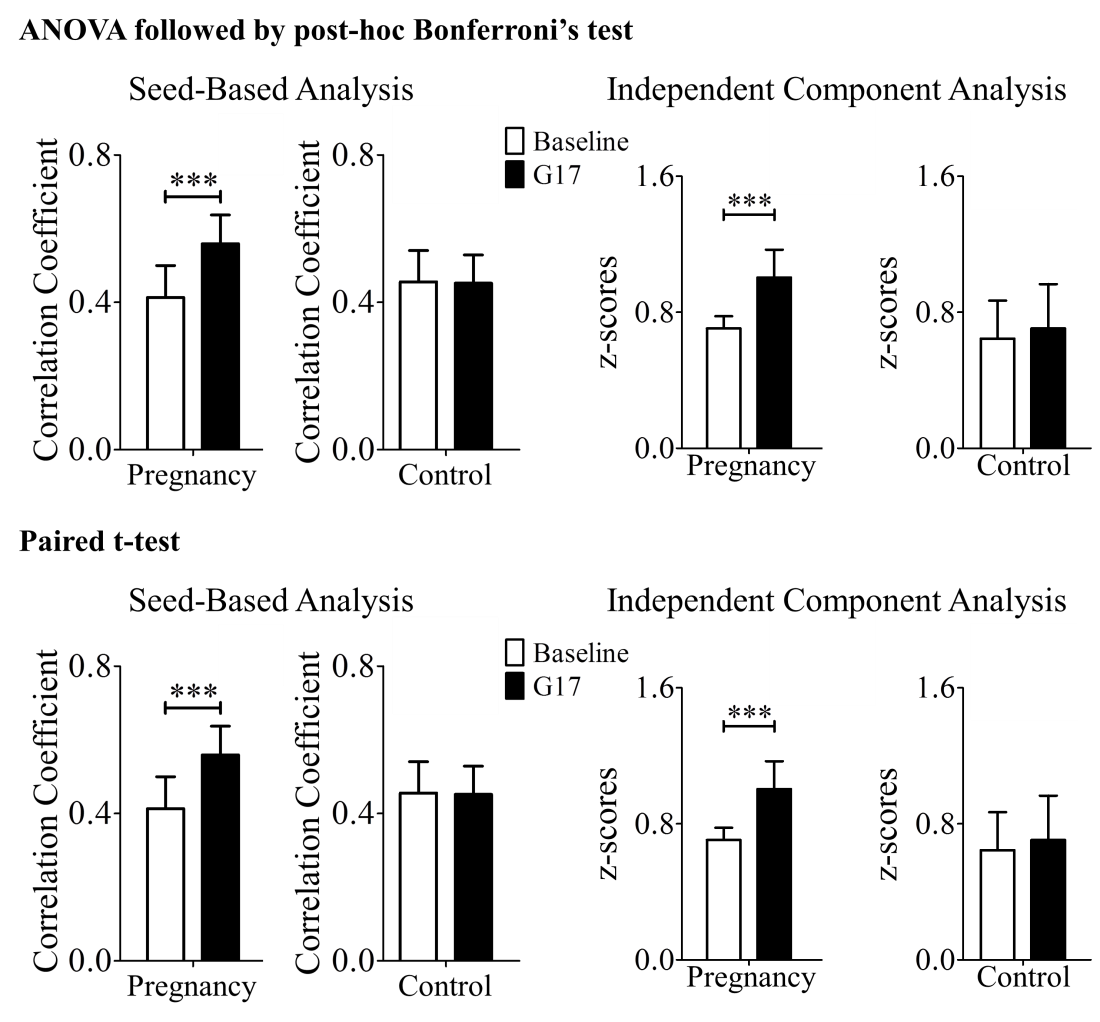


**Figure H.** Comparison between ANOVA followed by post-hoc Bonferroni’s test and paired t-test in bilateral hippocampal functional connectivity increase during pregnancy.

|  | Baseline | G17 |
| --- | --- | --- |
| Pregnancy Group (n=15) | $\frac{49 trials}{15 rats}=3.27 trials per rat$ | $\frac{52 trials}{15 rats}=3.47 trials per rat$ |
| Control Group (n=7) | $\frac{22 trials}{7 rats}=3.14 trials per rat$ | $\frac{23 trials}{7 rats}=3.29 trials per rat$ |

**Table A.** The average trials for the pregnancy and control groups at both baseline and G17 time-points used for subsequent seed-based and independent component analyses.
